# Brain Neurotransmitter Modulation by Gut Microbiota in Anxiety and Depression

**DOI:** 10.3389/fcell.2021.649103

**Published:** 2021-03-11

**Authors:** Fei Huang, Xiaojun Wu

**Affiliations:** Shanghai Key Laboratory of Compound Chinese Medicines, The Ministry of Education Key Laboratory for Standardization of Chinese Medicines, Shanghai R&D Center for Standardization of Chinese Medicines, Institute of Chinese Materia Medica, Shanghai University of Traditional Chinese Medicine, Shanghai, China

**Keywords:** serotonin, dopamine, noradrenaline, gut microbiota, anxiety- and depression-like behavior

## Abstract

Anxiety and depression are highly prevalent mental illnesses worldwide and have long been thought to be closely associated to neurotransmitter modulation. There is growing evidence indicating that changes in the composition of the gut microbiota are related to mental health including anxiety and depression. In this review, we focus on combining the intestinal microbiota with serotonergic, dopaminergic, and noradrenergic neurotransmission in brain, with special emphasis on the anxiety- and depression-like behaviors in stress-related rodent models. Therefore, we reviewed studies conducted on germ-free rodents, or in animals subjected to microbiota absence using antibiotics, as well as via the usage of probiotics. All the results strongly support that the brain neurotransmitter modulation by gut microbiota is indispensable to the physiopathology of anxiety and depression. However, a lot of work is needed to determine how gut microbiota mediated neurotransmission in human brain has any physiological significance and, if any, how it can be used in therapy. Overall, the gut microbiota provides a novel way to alter neurotransmitter modulation in the brain and treat gut–brain axis diseases, such as anxiety and depression.

## Introduction

Anxiety and depression are heterogeneous and complex diseases that can have devastating effects on the function and quality of life of individuals, and increase the risk of suicide (Arsenault-Lapierre et al., 2004). The overall burden of anxiety and depression is steadily increasing and now exceeds most other major diseases (Tyrovolas et al., 2020). Their onset can occur from childhood to adolescence and last a lifetime (Thapar and Riglin, 2020). In addition, anxiety and depression are often comorbid (Park and Kim, 2020) and relapse-prone conditions (Ali et al., 2017).

Despite the mechanisms of anxiety and depression are still unclear, neurotransmitters such as serotonin [also named 5-hydroxytryptamine (5-HT)], dopamine (DA), and noradrenaline (NE) have explained the pathophysiology of anxiety and depression over several decades (Olivier and Olivier, 2020; Shao and Zhu, 2020). An increasing number of evidence reveals the importance of the gut microbiota in the pathogenesis of anxiety and depression (Rieder et al., 2017). Gut microbiota and its metabolites are at least partially involved in the afferent input of the vagus nerve (Forsythe et al., 2014) and the regulation of the hypothalamic-pituitary-adrenal (HPA) axis (Sudo et al., 2004). Perhaps unsurprisingly, the gut microbiota has also been shown to be related to tryptophan metabolism and neurotransmitter production (Barrett et al., 2012; O’Mahony et al., 2015). Given the need to elucidate the potential role of gut microbiota in regulating neurotransmitter modulation in anxiety and depression, it seems important to summarize the evidence provided so far regarding the effects of neurotransmitters, in addition to uncovering behavioral alterations specific to these neurotransmitters.

## Methods

**FIGURE 1:**
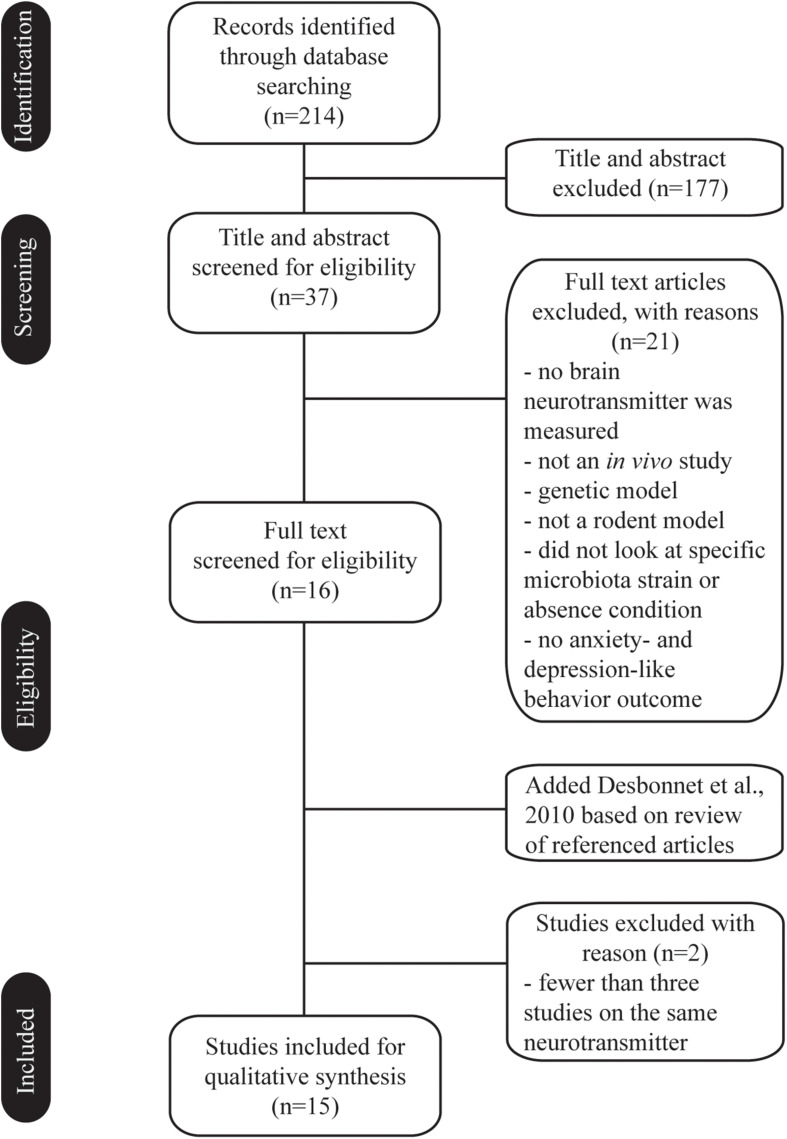
Flow chart of the selection process. Fifteen *in vivo* studies met the criteria and investigated the effect of gut microbiota in anxiety and depression, and in which brain neurotransmitters are part of the pathophysiology.

We searched for studies including text words related to microbiota (microbiome or flora) and neurotransmitter, and anxiety or depression. The search was carried out using the PubMed, Web of Science, and Embase databases. We included *in vivo* studies investigating gut microbiota in relation to anxiety and depression, and in which neurotransmitters are part of the pathophysiology, to facilitate comparisons. Studies excluded from the scope of search were or contained one or more of the following: did not use rodent species, did not look at the specific microbiota strain or absence condition, were not measuring anxiety- and depression-like behavior outcomes, no brain neurotransmitter was measured, did use genetic model, were not published in English. Additionally, in case there were fewer than three studies on the same neurotransmitter (e.g., gamma aminobutyric acid), these papers were also excluded due to lack of comparability. A total of 15 studies met the criteria for our review at the end of the selection process (Figure 1).

## Neurotransmitter Modulation and Behavioral Outcomes Identified Upon Modifications With Gut Microbiota

In this section, we summarize neurotransmitter parameter and behavior results identified in *in vivo* studies which used treatment with modifications by gut microbiota in the context of stress-related rodent models (Supplementary Table 1).

### Serotonin

5-Hydroxytryptamine is a neurotransmitter with important physiological significance in human body, involved in regulating many key processes, including behaviors, mood, gastrointestinal secretion, and peristalsis (Berger et al., 2009; Bamalan and Al Khalili, 2020). Antidepressants that act on 5-HT are utilized as front line drugs for many psychiatric disorders, such as major depressive disorder, post-traumatic stress disorder, anxiety, and bipolar disorder (Masand and Gupta, 1999; Bandelow et al., 2017). Although 5-HT is widely distributed throughout the body, 90–95% of 5-HT exists in the gastrointestinal tract (Gershon and Tack, 2007). Thus, it may not be surprising that the growing literature links the gut microbiota to host levels of 5-HT.

In germ-free (GF) rodents, two studies found no change of 5-HT and/or 5-hydroxyindoleacetic acid (5-HIAA) in the hippocampus and/or frontal cortex as well as in the striatum, but different anxiety-like behavioral alterations with early-life stress in C57BL/6N mice or acute stress in F344 male rats (Crumeyrolle-Arias et al., 2014; De Palma et al., 2015). Another two studies discovered reduced anxiety-like behavior in GF Swiss Webster mice, one in two reported higher 5-HT and 5-HIAA levels in the hippocampus of male mice, but no change of 5-HT and 5-HIAA levels in the hippocampus of female mice (Clarke et al., 2013), while another one found lower 5-HT receptor 1A (HTR1A) in the hippocampal DG rather than in the hippocampal CA1 of GF Swiss Webster female mice (Neufeld et al., 2011). In a model of antibiotic-induced depletion of the gut microbiota, one study revealed a greater display of depression-like behavior in Sprague–Dawley male rats. In tandem with the clear behavioral alteration, they also found lower 5-HT and higher 5-HIAA/5-HT in the hippocampus, and reduced 5-HIAA/5-HT in the hypothalamus (Hoban et al., 2016).

Seven studies investigated the antidepressant and/or anxiolytic effects of microbiota-based interventions on 5-HT modulation in anxiety and/or depression (Desbonnet et al., 2010; Liang et al., 2015; Sun et al., 2018; Li et al., 2019; Liao et al., 2019; Tian et al., 2019). Two studies detected live and heat-killed *Lactobacillus paracasei* PS23 in early life stress and corticosterone-treated models, respectively, but showed different results. For instance, both live and heat-killed *L. paracasei* PS23 treatment did not change 5-HT, 5-HIAA and 5-HIAA/5-HT in the hippocampus of early life stress induced model, while live *L. paracasei* PS23 treatment increased the level of 5-HT in the hippocampus and striatum of corticosterone-induced mice model rather than heat-killed PS23 treatment. In addition, both live and heat-killed *L. paracasei* PS23 treatment had no effect on the 5-HIAA in the prefrontal cortex and striatum of corticosterone-treated model. Besides that, probiotic *Bifidobacterium infantis* 35624 treatment resulted in reversal of depression-like behavioral deficits, but unchanged for 5-HIAA/5-HT in the hippocampus of early life stressed rat model. All the other five strains conducted by four studies were able to increase the brain 5-HT in stress-related rodent models. Regarding to specific brain area, *Lactobacillus helveticus* NS8, *Bifidobacterium longum*, and *Lactobacillus rhamnosus* increased the level of 5-HT in the hippocampus of rodents. Administration of *B. longum* subspecies *infantis* CCFM687, *B. longum* and *L. rhamnosus* showed higher 5-HT content in the frontal cortex. In particular, the study of *B. longum* subspecies *infantis* CCFM687 also found higher expression of 5-hydroxytryptophan (5-HPT) and no significant changes of HTR1A mRNA in the prefrontal cortex of male mice. In another study, chronic unpredictable mild stress-induced mice treated with *B. longum* and *L. rhamnosus* had higher tryptophan hydroxylase (TPH) but lower indoleamine 2,3-dioxygenase (IDO) in the prefrontal cortex and hippocampus compared with model group.

In the selected papers, only one study examined the effect of certain microbiota strain in GF mice, the behavior outcome showed live *Lactobacillus plantarum* PS128 had the anxiolytic effect along with higher 5-HT and 5-HIAA in the striatum without anti-depressant effects, rather the 5-HT, 5-HIAA, and 5-HIAA/5-HT in the prefrontal cortex and hippocampus as well as the 5-HIAA/5-HT in the striatum had no changes (Liu et al., 2016). In the same study, outcome of heat-killed *L. plantarum* PS128 tested in parallel has no statistical difference compared with the GF group.

The evidence summarized in this section highlights the role of gut microbiota in regulating 5-HT modulation in anxiety- and depression-like behavior in models of stress.

### Dopamine

Dopamine is the main catecholaminergic neurotransmitter, synthesized centrally and peripherally, that plays a pivotal role in multiple physiological processes such as emotion, memory, attention, motivation, reward, and food intake (Klein et al., 2019; Kleinridders and Pothos, 2019). DA system dysregulation has been related to anxiety (Carpenter et al., 2012; Moraga-Amaro et al., 2014), depression (Camardese et al., 2014; Belujon and Grace, 2017), and gut microbes (Gonzalez-Arancibia et al., 2019). In terms of crosstalk between gut and brain, the results (Han et al., 2018) strongly support that the vagus nerve is the key mediator.

Two studies observed the DA level in the hippocampus of GF rodents; both of them did not found significant change in comparison to specific pathogen-free (SPF) controls, although they reported inconsistent anxiety-like behavior under different stress (Crumeyrolle-Arias et al., 2014; De Palma et al., 2015). In GF F344 male rats, decreased DA, 3,4-dihydroxyphenylacetic acid (DOPAC), homovanillic acid (HVA), and HVA/DA were found in the frontal cortex, while reduced HVA and HVA/DA, an index of DA turnover, were reported in the hippocampus and striatum, but no statistic change of DA and DOPAC in the hippocampus and striatum was found. In a study using antibiotic-induced depletion of the gut microbiota in Sprague–Dawley male rats, they found higher levodopa (LDOPA) and HVA in the prefrontal cortex, lower HVA in the hippocampus, and lower HVA/DA in the amygdala and striatum. DA content had no significant changes in the above three brain regions compared to control group (Hoban et al., 2016).

Out of the four studies investigating the beneficial role of microbiota strain in DA system of depression models, all the three strains had capability to reduce depression- and/or anxiety-like behaviors. With respect to *L. paracasei* PS23 reported by two studies (Liao et al., 2019; Wei et al., 2019), both live and heat-killed *L. paracasei* PS23 administration decreased DOPAC and HVA, but not (DOPAC + HVA)/DA, in the hippocampus of early-life stress-induced male mice model. However, only heat-killed PS23 increased DA in the hippocampus and prefrontal cortex of corticosterone-treated mice model, and both live and heat-killed *L. paracasei* PS23 showed no effect on DOPAC in the prefrontal cortex of corticosterone-induced model. In the other two studies, one showed that DA had not changed significantly in the hippocampus and prefrontal cortex of chronic restraint stress-induced model after *L. helveticus* NS8 administration (Liang et al., 2015), while another study showed that treatment with *Bifidobacterium* CECT 7765 decreased DA level in the hypothalamus of early-life stress-induced male mice model (Moya-Perez et al., 2017).

In one study examined the effect of certain probiotic in GF mice, the behavior outcome showed that live *L. plantarum* PS128 exhibited the anxiolytic effect accompanying with higher DA and HVA in the striatum but not in the prefrontal cortex, hippocampus, the DOPAC and HVA/DA in the prefrontal cortex, hippocampus, and striatum did not change significantly (Liu et al., 2016). In the same study, outcome of heat-killed *L. plantarum* PS128 tested in parallel showed no effect on behavior and brain DA system in GF mice.

Based on the literatures summarized above, the potential of gut microbiota, to alleviate anxiety- and/or depression-like behavior, would take place via DA modulation.

### Noradrenaline

Noradrenaline has been known for its role in the pathogenesis of anxiety (Kalk et al., 2011; Zheng et al., 2019) and depression (Seki et al., 2018) for a long time. Interestingly, it appears NE also controls satiation (Asarian and Bachler, 2014). In addition, it has been reported that the microbiota influenced NE level in the gut lumen of mice (Asano et al., 2012), but whether the bacteria produce NE to alter behavior through an indirect path was not determined.

Noradrenaline modulation of GF rodents was measured in two studies, one in the context of increased anxiety-like behavior (Crumeyrolle-Arias et al., 2014) and another in the context of decreased depression-like behavior only in male mice under early-life stress (De Palma et al., 2015); in both conditions, NE was not affected in the brain. However, depletion of the microbiota with non-absorbable antibiotics has been reported to increase depression-like behavior; this effect was related to elevated level of NE in the striatum, but not in the prefrontal cortex, hippocampus, amygdala, or hypothalamus.

Administration of *L. helveticus* NS8, heat-killed *Enterococcus fecalis* (EC-12), and *Bifidobacterium* CECT 7765 improved anxiety-like behavior under different stress-related models (Liang et al., 2015; Moya-Perez et al., 2017; Kambe et al., 2020). NE is released via activation of central adrenoceptor β3 (Adrb3) (Claustre et al., 2008), while heat-killed *E. fecalis* (EC-12)-treated male mice expressed higher Adrb3 in the prefrontal cortex compared with control mice. In an early-life stress-induced model, *Bifidobacterium* CECT 7765 effectively reduced the content of NE in the hypothalamus of C57BL/6J male mice. Antidepressant effect was also demonstrated, in respect of *L. helveticus NS8* treatment showing elevated NE in the striatum but not in the prefrontal cortex of chronic restraint stress-induced male rats (Liang et al., 2015), whereas *B. infantis* 35624 treatment did not alter NE in the amygdaloid cortex of early-life stress-induced male rats (Desbonnet et al., 2010).

The limited evidence available on NE in anxiety- and depression-like behavior suggests that they could be influenced by gut microbiota.

Overall, modifications of gut microbiota can affect brain systems of 5-HT, DA, and NE in various rodent models of stress, as well as their anxiety- and depression-like behaviors.

## Discussion

This review summarizes the impacts of gut microbiota on the serotonergic, dopaminergic, and noradrenergic modulation in anxiety and depression. One way to address the effects of gut microbiota on the brain is to destroy the gut microbial ecology. Therefore, GF rodents provide a good tool to test the gut microbial colonization from early to adulthood (Gonzalez-Arancibia et al., 2019). While at first glance similar to the GF model, antibiotics represent another unique model to study the gut microbiome. Since destroying the microbiome can negatively affect the host, supplementation of the microbiome has been used as a strategy to optimize host performance. The introduction of known or suspected beneficial probiotic microorganisms is an intuitive way to study the relationship between the host and the microbiome. In this regard, it has been shown that levels of 5-HT, DA, and NE, and their respective precursor, metabolites, or receptors have significant variations across different brain regions in rodents with altered gut microbiota compared with their controls. However, it should be kept in mind that it may not only be a specific microbiota that is beneficial to microorganisms, but fundamentally provide nutrients that promote the growth of beneficial microorganisms (Duran-Pinedo and Frias-Lopez, 2015). A limitation of this review is that we did not include studies of prebiotics and fecal microbiota transplantation which are also general ways to alter microbiome composition, as there is little control over which microorganisms will metabolize or proliferate the prebiotics (Ford et al., 2014), and what kinds of fecal microbiota will be transplanted.

When considering how the bacteria may affect the brain neurotransmitters, one possible mechanism is that metabolite produced by the intestinal flora can be used as precursors for the synthesis of neurotransmitters in the central nervous system; for instance, *B. infantis* has been found to increase plasma tryptophan levels, thereby affecting the transmission of brain 5-HT (Desbonnet et al., 2010). Even though bacteria have been shown to have the capability to produce a series of major neurotransmitters including 5-HT, DA, and NE (Strandwitz, 2018), it is unlikely to influence brain directly because they cannot cross the blood–brain barrier. Besides, it must be taken into account that release of neurotransmitters is also regulated by other neural circuits (Russo and Nestler, 2013), and the influence of intestinal microbes on other networks cannot be excluded, which raises an open question: the regulation of brain neurotransmitters by gut microbes is direct, or indirect? Up to this review, this is still an unsolved question; moreover, the potential mechanisms of how the gut microbiota can affect the anxiety- and depression-like behavior via neurotransmitters are required to validate. Nevertheless, the studies reviewed indicated a close connection between intestinal symbionts and neurotransmitters in neuropsychiatric diseases, and it seems to be a possible way to communicate along the gut–brain axis. In addition, since most of the existing work has been done in animals, more well-designed human clinical trials are needed. Finally, more and more evidence supports that treatment for anxiety and depression could take advantage of intervention at the gut microbiota, either through reasonable use of antibiotics or via identification of novel microbial strains that influence the brain serotonergic, dopaminergic, and noradrenergic activity.

## Author Contributions

FH wrote the manuscript and researched the publication landscape. XW revised the manuscript and devised the idea. Both authors listed have made a substantial, direct, and intellectual contribution to the work, and approved it for publication.

## Conflict of Interest

The authors declare that the research was conducted in the absence of any commercial or financial relationships that could be construed as a potential conflict of interest.
