# Supplementary material for: Brain Neurotransmitter Modulation by Gut Microbiota in Anxiety and Depression
Source: Front Cell Dev Biol. 2021 Mar 11;9:649103. doi: 10.3389/fcell.2021.649103 (PMC7991717; doi:10.3389/fcell.2021.649103)
Supplement: Supplementary file 1 [file Table_1.docx]

**Supplementary Table 1 Behaviour and neurotransmitter outcomes identified upon modifications of gut microbiota.**

| **Modifications of gut microbiota** | **Strain and sex** | **Model** | **Behavior** | **Parameter** | **Reference** |
| --- | --- | --- | --- | --- | --- |
| **Serotonin (5-HT)** | | | | | |
| Absence (GF) | F344 male rats | Acute stress | Anxiety-like behavior ↗ | 5-HT (frontal cortex ns, hippocampus ns, striatum ns); 5-HIAA (frontal cortex ns, hippocampus ns, striatum ns) | (Crumeyrolle-Arias et al., 2014) * |
| Absence (GF) | C57BL/6N mice | Early-life stress | Anxiety-like behavior ns; depression-like behavior in male mice ↘; depression-like behavior in female mice ns | 5-HT (hippocampus ns) | (De Palma et al., 2015) * |
| Absence (GF) | Swiss Webster mice | Novel-environment stress | Anxiety-like behavior ↘ | 5-HT (hippocampus of male mice ↗, hippocampus of female mice ns), 5-HIAA (hippocampus of male mice ↗, hippocampus of female mice ns) | (Clarke et al., 2013) |
| Absence (GF) | Swiss Webster female mice | Acute stress | Anxiety-like behavior ↘ | HTR1A (hippocampal CA1 ns, hippocampal DG ↘) | (Neufeld et al., 2011) |
| Absence (ampicillin + vancomycin + ciprofloxacin HCl + imipenem + metronidazole treatment) | Sprague Dawley male rats | Acute stress | Anxiety-like behavior ns; depression-like behavior ↗ | 5-HT (prefrontal cortex ns, hippocampus ↘, amygdala ns, hypothalamus ns, striatum ns); 5-HIAA (prefrontal cortex ns, amygdala ns, hypothalamus ns, hippocampus ns, striatum ns); 5-HIAA/5-HT (prefrontal cortex ns, hippocampus ↗, amygdala ns, hypothalamus ↘, striatum ns) | (Hoban et al., 2016) * |
| *Lactobacillus helveticus* NS8 | Sprague Dawley male rats | Chronic restraint stress | Anxiety-like behavior ↘; depression-like behavior ↘ | 5-HT (hippocampus ↗) | (Liang et al., 2015) * |
| *Clostridium butyricum* | C57BL/6 male mice | Chronic unpredictable mild stress | Depression-like behavior ↘ | 5-HT (brain ↗) | (Sun et al., 2018) |
| *Bifidobacterium longum* | Wistar male rats | Chronic unpredictable mild stress | Depression-like behavior ↘ | 5-HT (frontal cortex ↗, hippocampus ↗); TPH2 (frontal cortex ↗, hippocampus ↗); IDO (frontal cortex ↘, hippocampus ↘) | (Li et al., 2019) |
| *Bifidobacterium infantis* 35624 | Sprague Dawley male rats | Early-life stress | Depression-like behavior ↘ | 5-HIAA/5-HT (hippocampus ns) | (Desbonnet et al., 2010) * |
| *Lactobacillus rhamnosus* | Wistar male rats | Chronic unpredictable mild stress | Depression-like behavior ↘ | 5-HT (frontal cortex ↗, hippocampus ↗); TPH2 (frontal cortex ↗, hippocampus ↗); IDO (frontal cortex ↘, hippocampus ↘); | (Li et al., 2019) |
| *Bifidobacterium longum* subspecies *infantis* strain CCFM687 | C57BL/6 male mice | Chronic stress | Anxiety-like behavior ↘; Depression-like behavior ↘ | 5-HT (prefrontal cortex ↗), 5-HTP (prefrontal cortex ↗), HTR1A mRNA (prefrontal cortex ns) | (Tian et al., 2019) |
| *Lactobacillus paracasei* PS23 (live and heat killed) | C57BL/6J male mice | Early-life stress | Anxiety-like behavior ↘; depression-like behavior ↘ | 5-HT (hippocampus ns), 5-HIAA (hippocampus ns), 5-HIAA/5-HT (hippocampus ns) | (Liao et al., 2019) * |
| *Lactobacillus paracasei* PS23 (live and heat killed) | C57BL/6J male mice | Corticosterone treatment | Anxiety-like behavior ↘, depression-like behavior ↘ | 5-HT (hippocampus of live bacteria treatment ↗, hippocampus of heat killed bacteria treatment ns, striatum of live bacteria treatment ↗, striatum of heat killed bacteria treatment ns ); 5-HIAA (prefrontal cortex ns, striatum ns) | (Wei et al., 2019) * |
| *Lactobacillus plantarum* PS128 (live and heat killed) + Absence (GF) | C57BL/6JNarl male mice | Acute stress | Anxiety-like behavior (live)↘; anxiety-like behavior (heat killed) ns; depression-like behavior ns | 5-HT (prefrontal cortex ns, hippocampus ns, striatum of live bacteria treatment ↗, striatum of heat killed bacteria treatment ns); 5-HIAA (prefrontal cortex ns, hippocampus ns, striatum of live bacteria treatment ↗, striatum of heat killed bacteria treatment ns); 5-HIAA/5-HT (prefrontal cortex ns, hippocampus ns, striatum ns) | (Liu et al., 2016) * |
| **Dopamine (DA)** | | | | | |
| Absence (GF) | F344 male rats | Acute stress | Anxiety-like behavior ↗ | DA (frontal cortex ↘, hippocampus ns, striatum ns); DOPAC (frontal cortex ↘, hippocampus ns, striatum ns); HVA (frontal cortex ↘, hippocampus ↘, striatum ↘); HVA/DA (frontal cortex ↘, hippocampus ↘, striatum ↘) | (Crumeyrolle-Arias et al., 2014) * |
| Absence (GF) | C57BL/6N mice | Early-life stress | Anxiety-like behavior ns; depression-like behavior in male mice ↘; depression-like behavior in female mice ns | DA (hippocampus ns) | (De Palma et al., 2015) * |
| Absence (ampicillin + vancomycin + ciprofloxacin HCl + imipenem + metronidazole treatment) | Sprague Dawley male rats | Acute stress | Anxiety-like behavior ns, depression-like behavior ↗ | DA (prefrontal cortex ns, amygdala ns, striatum ns); LDOPA (prefrontal cortex ↗, hippocampus ↗); HVA (prefrontal cortex ↗, hippocampus ↘, amygdala ns, hypothalamus ns, striatum ns), HVA/DA (prefrontal cortex ns, amygdala ↘, striatum ↘) | (Hoban et al., 2016) * |
| *Lactobacillus helveticus* NS8 | Sprague Dawley male rats | Chronic restraint stress | Anxiety-like behavior ↘, depression-like behavior ↘ | DA (prefrontal cortex ns, hippocampus ns) | (Liang et al., 2015) * |
| *Lactobacillus paracasei* PS23 (live and heat killed) | C57BL/6J male mice | Early-life stress | Anxiety-like behavior ↘, depression-like behavior ↘ | DA (hippocampus ns); DOPAC (hippocampus ↘); HVA (hippocampus ↘); (DOPAC+HVA)/DA (hippocampus ns) | (Liao et al., 2019) * |
| *Lactobacillus paracasei* PS23 (live and heat killed) | C57BL/6J male mice | Corticosterone treatment | Anxiety-like behavior ↘, depression-like behavior ↘ | DA (hippocampus of live bacteria treatment ns, hippocampus of heat killed bacteria treatment ↗, prefrontal cortex of live bacteria treatment ns, prefrontal cortex of heat killed bacteria treatment ↗); DOPAC (prefrontal cortex ns) | (Wei et al., 2019) * |
| *Bifidobacterium* CECT 7765 | C57BL/6J male mice | Early-life stress | Anxiety-like behavior ↘ | DA (hypothalamus ↘) | (Moya-Perez et al., 2017) * |
| *Lactobacillus plantarum* PS128 (live and heat killed) + Absence (GF) | C57BL/6JNarl male mice | Acute stress | Anxiety-like behavior (live)↘; anxiety-like behavior (heat killed) ns; depression-like behavior ns | DA (prefrontal cortex ns, hippocampus ns, striatum of live bacteria treatment ↗, striatum of heat killed bacteria treatment ns); DOPAC (prefrontal cortex ns, hippocampus ns, striatum ns); HVA (prefrontal cortex ns, hippocampus ns, striatum of live bacteria treatment ↗, striatum of heat killed bacteria treatment ns); HVA/DA (prefrontal cortex ns, hippocampus ns, striatum ns) | (Liu et al., 2016) * |
| **Noradrenaline (NE)** | | | | | |
| Absence (GF) | F344 male rats | Acute stress | Anxiety-like behavior ↗ | NE (frontal cortex ns, hippocampus ns, striatum ns) | (Crumeyrolle-Arias et al., 2014) * |
| Absence (GF) | C57BL/6N mice | Early-life stress | Anxiety-like behavior ns; depression-like behavior in male mice ↘; depression-like behavior in female mice ns | NE (hippocampus ns) | (De Palma et al., 2015) * |
| Absence (ampicillin + vancomycin + ciprofloxacin HCl + imipenem + metronidazole treatment) | Sprague Dawley male rats | Acute stress | Anxiety-like behavior ns Depression-like behavior ↗ | NE (prefrontal cortex ns, hippocampus ns, amygdala ns, hypothalamus ns, striatum ↗) | (Hoban et al., 2016) * |
| *Lactobacillus helveticus* NS8 | Sprague Dawley male rats | Chronic restraint stress | Anxiety-like behavior ↘, depression-like behavior ↘ | NE (prefrontal cortex ns, hippocampus ↗) | (Liang et al., 2015) * |
| Heat killed *Enterococcus fecalis* (EC-12) | C57BL/6 male mice | Acute stress | Anxiety-like behavior ↘ | Adrb3 (prefrontal cortex ↗) | (Kambe et al., 2020) |
| *Bifidobacterium* CECT 7765 | C57BL/6J male mice | Early-life stress | Anxiety-like behavior ↘ | NE (hypothalamus ↘) | (Moya-Perez et al., 2017) * |
| *Bifidobacterium infantis* 35624 | Sprague Dawley male rats | Early-life stress | Depression-like behavior ↘ | NE (amygdaloid cortex ns) | (Desbonnet et al., 2010) * |

5-HIAA, 5-hydroxyindoleacetic acid; 5-HT, 5-hydroxytryptamine; 5-HTP, 5-Hydroxytryptophan; Adrb3, adrenoceptor β3; DA, dopamine; DOPAC, 3,4-Dihydroxyphenylacetic acid; GF, germ free; HTR1A, 5-Hydroxytryptamine receptor 1A; HVA, homovanillic acid; IDO, indoleamine 2,3-dioxygenase; LOPA, levodopa; NE, noradrenaline; TPH, tryptophan hydroxylase.

↗, increase; ↘, decrease; ns, no statistical difference; *, article appearing several times.
